# Polymorphisms associated with a tropical climate and root crop diet induce susceptibility to metabolic and cardiovascular diseases in Solomon Islands

**DOI:** 10.1371/journal.pone.0172676

**Published:** 2017-03-02

**Authors:** Takuro Furusawa, Izumi Naka, Taro Yamauchi, Kazumi Natsuhara, Ricky Eddie, Ryosuke Kimura, Minato Nakazawa, Takafumi Ishida, Ryutaro Ohtsuka, Jun Ohashi

**Affiliations:** 1 Graduate School of Asian and African Area Studies, Kyoto University, Kyoto, Japan; 2 Graduate School of Science, The University of Tokyo, Tokyo, Japan; 3 Graduate School of Health Sciences, Hokkaido University, Hokkaido, Japan; 4 The Japanese Red Cross Akita College of Nursing, Akita, Japan; 5 National Gizo Hospital, Ministry of Health and Medical Services, Gizo, Solomon Islands; 6 Graduate School of Medicine, University of the Ryukyus, Okinawa, Japan; 7 Department of International Health, Kobe University Graduate School of Health Sciences, Kobe, Japan; 8 Japan Wildlife Research Center, Tokyo, Japan; Jichi Medical University, JAPAN

## Abstract

The people of the Solomon Islands represent an Austronesian (AN)-speaking population’s adaptation to a humid tropical environment and subsistence of tuberous crops. Genome-wide association studies (GWASs) of other populations (e.g. the Human Genome Diversity Project [HGDP]) have suggested the existence of genotypes adaptive to ecoregion, diet, and subsistence, and that those genotypes are also associated with metabolic and cardiovascular diseases. Recently, the incidence of non-communicable diseases has been increasing in the Solomon Islands. In the present study, we explored the association of genotypes adaptive to a tropical environment and tuberous crop diet with metabolic and cardiovascular conditions in rural and urban AN-speaking Melanesian and Micronesian populations of the Solomon Islands. A total of 561 participants were genotyped for single nucleotide polymorphisms (SNPs) potentially associated with a tropical environment (rs174570 and rs2237892) and a tuberous crop diet (rs162036, rs185819, and rs2722425). The results showed that the allele frequencies of the Solomon Islands populations adopted patterns similar to those in populations from other hot, tropical areas with a tuberous crop diet in previous studies. Furthermore, rs162036, rs185819, rs2237892, and rs2722425 were all strongly associated with one or more metabolic and cardiovascular conditions. The derived allele of rs2722425 (i.e. rs2722425-G) was significantly associated with an elevated LDL level (*P* = 0.000264) even after the significance level was adjusted for multiple testing (i.e., *α* = 0.0005). Our results suggest that the inhabitants of the Solomon Islands exhibit the effects of the tropical environment and tuberous crop diet on their allele frequencies, and that their susceptibility to metabolic and cardiovascular diseases is therefore considered to be associated with their environment and diet.

## Introduction

Located in Melanesia, the Solomon Islands is a country with a humid tropical climate. For example, New Georgia Island, the biggest island in Western Province, is covered by dense tropical forest with average maximum and minimum temperatures of 30.3 and 24.2°C, respectively, and a mean annual rainfall of 3458 mm (recorded at Munda in 1993) [1]. The people living on these islands have traditionally depended on shifting cultivation of tuberous crops, such as taro and yams, although their main crops have shifted to sweet potato and cassava in the last century, and many have adopted a Westernized diet of rice in the past decades. Much attention has been focused on the genetic background of Solomon Islands people because the Pacific Islands is a region of the world that has only lately been occupied by humans. Although there is still debate on the origin and migration route of the Pacific Island people [2–4], linguistic, archaeological, and genetic studies have suggested that the Austronesian (AN)-speaking populations originated from East Asia (or Southeast Asia in some studies), passed through the islands by voyaging, and arrived in Melanesia about 1500 years BCE, around the Gilbert Islands of Micronesia 500 years BCE, and in Polynesia by 1000 years CE [5]. The majority of the people in New Guinea and the nearby islands are Non-Austronesian (NAN)-speaking and descendants of the first human populations who settled in this region but did not migrate further, likely because they had not yet developed the technology required for long voyaging. Today, the Solomon Islands consists of AN-speaking Melanesians (as the majority), NAN-speaking Melanesians, AN-speaking Polynesian outliers, immigrant AN-speaking Micronesians, and others.

The ‘thrifty genotype’ of the Pacific Islands populations has been the subject of substantial debate [6]. Under this hypothesis, the genes that predispose individuals to obesity or high carbon storage would have had a selective advantage in populations that frequently experienced starvation (such as on prehistoric inter-island voyages), but individuals with this genotype are necessarily susceptible to severe weight gain or diabetes when a stable food source is available. Another thrifty genotype hypothesis concerns their elevated blood pressure and salt avidity. Enhanced salt and water avidity and vascular reactivity are adaptive traits associated with hot and humid environments; however, such individuals are at risk of high blood pressure when salt is readily available [7–9].

In the Solomon Islands, as well as several other Pacific Islands societies, the incidence of disorders related to obesity, glucose metabolism, and the cardiovascular function has been increasing recently, and non-communicable disease are now a more urgent issue than infectious diseases and malnutrition [10–12]. In our previous studies, the presence of the high-risk genotypes found in European or Asian populations was evaluated among populations in the Solomon Islands. Significant associations observed for the leptin receptor (*LEPR*) gene and angiotensinogen (*AGT*) gene polymorphisms with obesity and hypertension, respectively, supported the notion that the prevalence of high-risk alleles increased with adaptation to an island environment. However, risk alleles for the fat mass and obesity associated (*FTO*) gene and leptin (*LEP*) gene, which are associated with obesity in European populations, were not associated with obesity in the Solomon Islands populations [7, 13]. In addition, the hypertension-susceptibility genes of G-protein β3-subunit (*GNB3*) and cytochrome P450 3A5 (*CYP3A5*) were not associated with elevated blood pressure in Solomon Islands populations [9]. These results suggest that disease susceptibility genes in European and Asian populations are not always risk-related genotypes in Solomon Islands populations, but environment-related genotypes may be associated with certain disease risks in these populations.

Previous genome-wide association studies (GWASs) of populations sampled worldwide—but not including the Solomon Islands or other AN-speaking populations—have found gene polymorphisms representing human adaptation to climate, diet, and subsistence, and some of these polymorphisms were also found to be associated with increased risks for certain metabolic and cardiovascular diseases [14, 15]. In the present study, we analyzed the allele frequencies of gene polymorphisms that have been found to be related to humid tropical climates and a root-crop diet, representing the environment and lifestyle of two AN-speaking Melanesian populations living in rural and urban settings in the Solomon Islands and one AN-speaking Micronesian population who migrated from their original location in the 1960s for political reasons. We also explored the association of genotypes adapted to this environment and diet with metabolic and cardiovascular diseases.

## Materials and methods

### Study populations

The study participants were recruited from three populations in the Solomon Islands: (1) Kusaghe in North New Georgia, (2) Munda Town in Southwestern New Georgia; and (3) Micronesians in Ravaki Village in Western Province on Kohinggo Island (opposite shore of New Georgia Island). Although Ravaki Village is located in the Solomon Islands, the inhabitants migrated there from the overpopulated Gilbert Islands (Kiribati) in the 1960s.

A total of 215, 187, and 166 adult subjects (≥ 18 years old) were recruited from Kusaghe, Munda Town, and Ravaki Village, respectively. Thirteen of the Ravaki participants were excluded from the analyses because one or both parents was not a Micronesian migrant (e.g. Melanesians who entered Ravaki Village through marriage). Four of the Munda participants were excluded because one or both parents was not Melanesian (e.g. Micronesian or European). All of the Kusaghe participants were of Melanesian descent, so none was excluded. In total, 561 subjects were analyzed in this study. It should be noted that the population size at each study site was relatively small (approximately 1000, 3000, and 800 in Kusaghe, Munda, and Ravaki, respectively, including all age groups), all study villages were neither geographically nor socially isolated and thus people in these villages were connected with other villages through intermarriage in New Georgia Island and other islands in the Western Province. The Ravaki people were also connected with other Micronesian communities in the province (e.g., in Gizo Island).

All measurements and sampling were performed after obtaining informed consent (written informed consent) from each participant. This study was approved by the Health Research Ethics Committee, the Ministry of Health and Medical Services of the Solomon Islands, and the Research Ethics Committee of the Faculty of Medicine and Faculty of Science at the University of Tokyo, Japan.

### SNPs analyzed in this study

Blood was sampled in the field, and genomic DNA was extracted using the QIAamp Blood Kit (Qiagen, Hilden, Germany). All five SNPs (rs162036, rs174570, rs185819, rs2237892, and rs2722425) were genotyped using the TaqMan^®^ SNP genotyping assay (Applied Biosystems, Carlsbad, CA, USA).

Rs162036 (ancestral = A; derived = G) is a genomic SNP of missense variation (K [Lys] > R [Arg]) of 5-methyltetrahydrofolate-homocysteine methyltransferase reductase (*MTRR*) at a location 5p15.31 and plays a key role in the conversion of homocysteine (Hcy) to methionine. This gene functions in dietary folate metabolism ancestrally, and a more derived allele has been found in societies consuming root vegetables and tubers (i.e. a folate-poor diet) [16]. It has also been reported that a vegetarian diet (deficient in vitamin B_12_) and genetic factors can cause hyperhomocysteinemia in Indian populations [17], and hyperhomocysteinemia is a risk factor for cardiovascular diseases. In addition, rare mutations in this and other SNPs in *MTRR* have been associated with the plasma Hcy level and thus are found relatively frequently in type 2 diabetes mellitus patients among Han Chinese [18].

Rs174570 (ancestral = C; derived = T) is a genomic SNP located in the intron region of fatty acid desaturase 2 (*FADS2*) at 11q12.2. The derived allele of this SNP is found more frequently in subjects living in a humid tropical ecoregion than in others [15], and high differentiation between northern and southern groups within Han Chinese [19] and high geographic variation [20] have also been reported. This gene plays a key role in polyunsaturated fatty acid (PUFA) metabolism and has been linked to adipose tissue formation and cardiovascular risk. The ancestral allele (C) of this SNP is associated with elevated total cholesterol, high-density lipoprotein (HDL), low-density lipoprotein (LDL), and decreased triglyceride (TG) levels in European populations [21]; elevated HDL levels in global populations [16]; and elevated linoleic acid (LA) and alpha linoleic acid (ALA) and decreased arachidonic acid (AA) levels in European, Hispanic, and Japanese populations [22–26].

Rs185819 (ancestral = C; derived = T) is a genomic SNP of the missense function (H [His] > R [Arg]) of tenascin XB (*TNXB*) at 6p21.33-p21.32. Hancock found that the derived allele (T) of this SNP is more prevalent in populations living in regions with a high maximum summer temperature [16]. This SNP is involved in the collagen metabolic process [27] and is associated with height in some studies [28–30] but was not found to be associated with height in an Australian twin families study [31] or in a Northern Finland birth cohort [32]. Rs2237892 (ancestral = C; derived = T) is a genomic SNP located in the intron region of a potassium voltage-gated channel, KQT-like subfamily, member 1 (*KCNQ1*) at 11p15.5-p15.4. Hancock reported that a more ancestral allele was found in societies subsisting on cereal grains [16], an unfamiliar diet style in the Solomon Islands. The ancestral allele (C) has been reported to be associated with an increased risk of type 2 diabetes among Korean, Chinese, Japanese, Indian, and European populations [33–35] and in a global meta-analysis [36, 37]; this allele was also associated with higher fasting glucose levels in a Chinese population [38], coronary artery disease (CAD) [39], and a higher body mass index in diabetes patients [40]. In contrast, the T allele was associated with decreased TG levels in a Han population [41].

Finally, rs2722425 (ancestral = G; derived = A) is a genomic SNP located in the intron region of a zinc finger, matrin-type 4 (*ZMAT4*) at 8p11.21. A previous study showed that the derived allele of this SNP is more prevalent in societies consuming root vegetables and tubers and is associated with fasting plasma glucose levels [16], although very few studies have analyzed this SNP.

### Human genome diversity project data

The Stanford HGDP SNP genotyping data (N = 1043), which are publicly available online (http://www.hagsc.org/hgdp/), were analyzed to compare the allele frequencies of East Asian, European, Melanesian (NAN-speaking), Middle Eastern, North African, Latin American, South Asian, Southeast Asian, and Sub-Sahara African populations with Solomon Islands populations in this study.

### Anthropometric, blood pressure measurements, and blood biochemical analyses

All participants were asked to arrive to the survey center (hospital and/or community hall) before 0900 hours. Body height was measured to the nearest 1 mm using a field anthropometer (GPM, Zurich, Switzerland), and weight was recorded to the nearest 0.1 kg using a portable digital scale (BC-518; Tanita, Tokyo, Japan), in accordance with a previously reported protocol [7]. Participants with BMI ≥25 kg/m^2^ were classified as overweight [42].

The sitting blood pressure was measured twice from the arm using a digital blood pressure monitor (HEM757; Omron, Kyoto, Japan); the average of the two values was used. Hypertension was defined as ≥140 mmHg for systolic blood pressure (SBP) and/or ≥90 mmHg for diastolic blood pressure (DBP). Of the 399 participants, 16 were pregnant or had already been diagnosed with hypertension and treated with antihypertensive drugs; these participants were excluded from subsequent analyses.

In addition, fasting blood was drawn by the staff of the National Gizo Hospital from the antecubital vein in the early morning. The serum was then separated and immediately frozen. The samples were stored at -20°C and transported to Japan. The serum leptin concentration, serum glucose (indicator of diabetes), total cholesterol (indicator of risk of hypercholesterolemia and arterial sclerosis), LDL, and HDL levels were measured by Kaketsuken (the Chemo-Sero-Therapeutic Research Institute) in Kumamoto, Japan, using the double-antibody-type radioimmunoassay (RIA) method (Human Leptin RIA kit; Linco Research Inc., St. Charles, MO, USA); the limit of sensitivity of this kit is 0.5 ng/mL. Quality control measurements and the differences between duplicate results were well within the expected ranges outlined by the manufacturer. Serum glucose ≥110 mg/dL, serum cholesterol ≥240 mg/dL, and serum LDL ≥140 mg/dL were defined as diabetes, high cholesterol, and high LDL, respectively.

### Statistical analyses

The goodness of fit *χ*^2^ was used to test for Hardy-Weinberg equilibrium (HWE) for each SNP in each population. An analysis of covariance (ANCOVA) was performed to compare the body weight and height, BMI, and serum cholesterol, serum LDL, serum HDL, and serum leptin levels between homozygotes of ancestral alleles and carrier genotypes of the derived allele, and a multiple logistic regression analysis was performed to compare the occurrence of overweight, diabetes, hypertension, high cholesterol, and high LDL between homozygotes of ancestral alleles and carrier genotypes of the derived allele; adjustments were made for gender (female = 1, male = 0), age (years), and population differences (dummy variables) in all of these analyses. *P* <0.05 was considered statistically significant in each analysis, *P* <0.0005 was considered statistically significant for the ANCOVA after Bonferroni’s adjustment (10 phenotypes by five SNPs by two comparisons), and *P* <0.001 was considered statistically significant for the multiple logistic regression analysis (five phenotypes by five SNPs by two comparisons). All statistical analyses were performed using the R software program, ver. 3.1.3 (R Project) with the ‘genetics’ extension.

## Results

**Fig 1:**
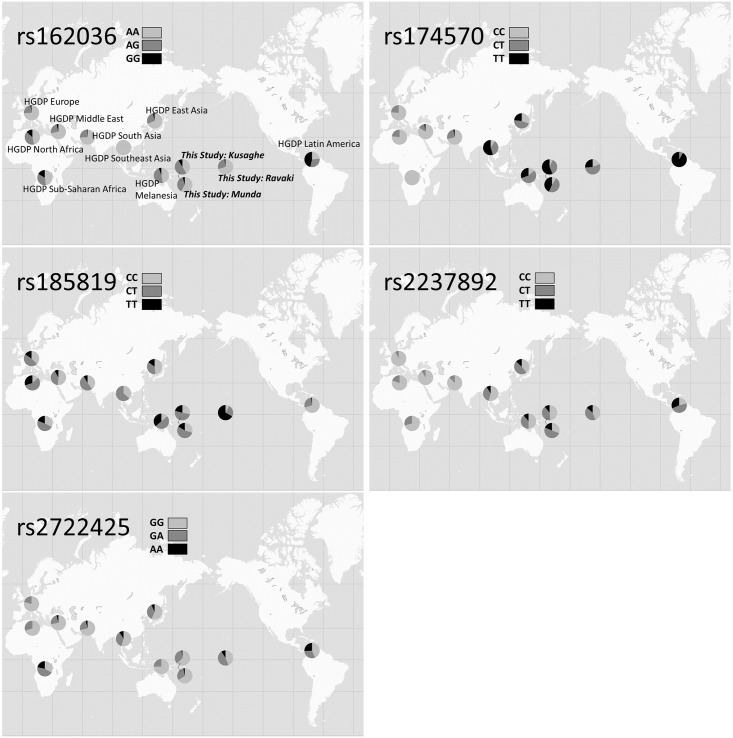
The allele frequencies of five SNPs in populations analyzed in the Human Genome Diversity Project (HGDP) and in this study. This study analyzed the Kusaghe (rural Melanesian, N = 183), Munda (urban Melanesian, N = 215), and Ravaki peoples (Micronesian, N = 163); Ravaki people were sampled from the Solomon Islands, and their original location is shown on the map. HGDP East Asia (N = 234) includes the following geographic origins: China, Japan, Siberia; HGDP Europe (N = 160): France, Italy, Italy (Bergamo), Orkney Islands, Russia, Russia (Caucasus); HGDP Melanesia (N = 36): Bougainville, Papua New Guinea, HGDP Middle East (N = 146): Israel (Carmel), Israel (Central), Israel (Negev); HGDP North Africa (N = 30): Algeria (Mzab); HGDP Latin America (N = 108): Brazil, Colombia, and Mexico; HGDP South Asia (N = 197): Pakistan; HGDP Southeast Asia (N = 11): Cambodia; and HGDP Sub-Saharan Africa (N = 121): Central African Republic, Democratic Republic of Congo, Kenya, Namibia, Nigeria, Senegal, and South Africa.

Genotyping showed no significant deviations from the Hardy-Weinberg equilibrium for any SNP in any population. Fig 1 and S1 Table show the allele frequencies of the study population in the Solomon Islands and the HGDP; detailed frequencies for the former are also shown in Table 1. When the proportions of the derived alleles were compared among populations, the proportion of G carriers of rs162036 was higher in the Solomon Islands populations (root crop diet) than in the populations of Asia (East, Southeast, and South Asia), the Middle East, and Europe, which are less dependent on root vegetable crops than Pacific Islanders. The proportion of the derived allele (T) of rs174570 in the Solomon Islands populations was as high as in other humid tropical regions, such as Southeast Asia and Latin America, although this allele was rare in tropical Africa. The proportion of the derived allele (T) of rs185819 was as high in the Solomon Islands populations as in other high-temperature areas (North and Sub-Saharan Africa and Melanesia). Furthermore, the proportion of the homozygote TT type was very high in Ravaki Village. Although a previous study reported that the proportions of the ancestral type (C) of rs2237892 were high in cereal-dependent societies [16], the proportion of the C type was low in the Solomon Islands populations; however, this proportion was as high as in cereal-dependent populations of East and Southeast Asia. Finally, unexpectedly, in the Solomon Islands populations, the proportion of the derived type (A) of rs2722425 was not as high as in the root crop diet populations of Latin America and North Africa; in the HGDP data, the proportion of the A type was also low in the root crop diet populations of Melanesia (NAN-speaking).

**Table 1:** Characteristics of study participants, broken down by populations.

		Kusaghe (Rural, Melanesian) N = 215	Munda (Urban, Melanesian) N = 183	Ravaki (Peri-urban, Micronesian) N = 153
Gender (% male)		106:109 (49.3%)	91:95 (48.9%)	57:61 (48.3%)
Age (years)		37.17 ± 0.93	45.43 ± 1.06	34.62 ± 1.21
Body height (cm)		159.23 ± 0.47	159.60 ± 0.55	165.27 ± 0.77
Body weight (kg)		60.97 ± 0.49	65.88 ± 1.01	79.13 ± 1.51
BMI (kg/m^2^)		24.06 ± 0.18	25.84 ± 0.37	28.96 ± 0.51
SBP (mmHg)		119.38 ± 0.97	127.13 ± 1.56	117.20 ± 1.26
DBP (mmHg)		71.16 ± 0.64	79.22 ± 0.90	76.32 ± 0.96
Total cholesterol (mg/dL)		179.63 ± 2.37	185.85 ± 2.74	166.17 ± 3.50
LDL (mg/dL)		113.73 ± 2.05	126.22 ± 2.43	114.64 ± 3.14
HDL (mg/dL)		51.08 ± 0.76	43.56 ± 0.76	39.84 ± 0.88
Glucose (mg/dL)		94.54 ± 2.67	94.86 ± 1.47	98.45 ± 2.84
Leptin (mg/dL)		6.86 ± 0.44	13.52 ± 1.00	10.73 ± 0.83
Obesity (BMI ≥ 30 kg/m^2^)		2.4%	18.6%	30.1%
Hypertension (SBP ≥ 140 mmHg and/or DBP ≥ 90 mmHg)		8.6%	23.0%	12.3%
Diabetes (glucose ≥ 110 mg/dl)		7.0%	8.1%	17.8%
High total cholesterol (cholesterol ≥ 240 mg/dl)		5.1%	8.1%	1.7%
High LDL (LDL ≥ 140 mg/dl)		18.6%	29.2%	22.9%
Allele Frequencies				
Rs162036	AA	41.4%	58.5%	70.1%
	AG	49.8%	36.6%	28.8%
	GG	8.8%	4.9%	2.4%
Rs174570	CC	7.0%	9.3%	18.9%
	CT	36.7%	47.5%	54.3%
	TT	56.3%	43.2%	26.2%
Rs185819	CC	27.9%	29.5%	7.9%
	CT	51.2%	54.1%	25.0%
	TT	20.9%	16.4%	66.5%
Rs2237892	CC	48.4%	30.6%	44.5%
	CT	40.9%	50.8%	41.5%
	TT	10.7%	18.6%	14.0%
Rs2722425	GG	62.8%	66.1%	43.9%
	GA	35.3%	30.6%	47.0%
	AA	1.9%	3.3%	9.1%

BMI, body mass index; DBP, diastolic blood pressure; HDL, high-density lipoprotein; LDL, low-density lipoprotein; SBP, systolic blood pressure.

Table 1 shows the characteristics of the study participants, broken down by populations. Residents of Munda Town (urban Melanesian) and Ravaki Village (peri-urban Micronesian) were more likely to be obese than those in Kusaghe (rural Melanesian). Obesity was more prevalent in urban Melanesian (Munda Town) and peri-urban Micronesian (Ravaki Village) populations than in rural Melanesian ones. High blood pressure, hypercholesterolemia, and high LDL were more frequent in urban Melanesia than in other regions, while diabetes was more frequent in Micronesia than in other regions.

Table 2 shows the effects of the genotypes on health indicators; the coefficients for the confounding variables and model *R*^2^ values are shown in S2–S8 Tables. The AG type of the rs162036 increased the BMI by 0.80 kg/m^2^ (*P* = 0.04190) and the SBP by 4.48 mmHg (*P* = 0.00279) compared with the AA after adjusting for age, sex, and population differences; the GG type showed no significant difference from the AA type. The TT type of rs185819 significantly increased the BMI by 1.21 kg/m^2^ (*P* = 0.040749) and the serum leptin (*P* = 0.02253); the effect on leptin was manifested only in females when analyzed separately for respective genders (S8 Table). In addition, this type decreased the total cholesterol by -9.39 mg/dL (*P* = 0.0395) and LDL by -8.04 mg/dL (*P* = 0.048), and the serum glucose by -10.06 mg/dL (*P* = 0.01805). The TT type of rs2237892 increased the height by 1.62 cm (*P* = 0.0286) and DBP by 3.20 mmHg (*P* = 0.021); the effects on the height disappeared when the analyses were made separately for the respective genders (S7 Table). The GA type and AA type of rs2722425 significantly increased the total cholesterol by 10.44 mg/dL (*P* = 0.00065) and 14.36 mg/dL (*P* = 0.046), respectively, and the GA type increased the LDL by 9.98 mg/dL (*P* = 0.000264), which was more than the GG type. The effect of the GA type on the LDL was only significant after Bonferroni’s adjustment.

**Table 2:** The effects of tropical-climate-related SNPs and root-crop-diet-related SNPs on health indicators; general linear model, after adjustment for age, gender, and population differences (also see S2–S6 Tables).

	rs162036		rs174570		rs185819		rs2237892		rs2722425	
Body height (cm)	AA vs. AG	− 0.20 (0.51) NS	CC vs. CT	1.09 (0.79) NS	CC vs. CT	0.77 (0.62) NS	CC vs. CT	0.75 (0.52) NS	GG vs. GA	− 0.43 (0.51) NS
	AA vs. GG	− 0.71 (1.04) NS	CC vs. TT	1.11 (0.82) NS	CC vs. TT	0.11 (0.75) NS	CC vs. TT	1.62 (0.74) *P* = 0.0286	GG vs. AA	− 0.37 (1.20) NS
Body weight (kg)	AA vs. AG	2.00 (1.15) NS	CC vs. CT	0.54 (1.80) NS	CC vs. CT	1.59 (1.40) NS	CC vs. CT	1.54 (1.17) NS	GG vs. GA	0.23 (1.15) NS
	AA vs. GG	− 3.06 (2.33) NS	CC vs. TT	1.28 (1.86) NS	CC vs. TT	2.97 (1.70) NS 0	CC vs. TT	2.69 (1.68) NS	GG vs. AA	− 0.63 (2.71) NS
BMI (kg/m^2^)	AA vs. AG	0.80 (0.40) *P* = 0.04190	CC vs. CT	− 0.13 (0.62) NS	CC vs. CT	0.38 (0.48) NS	CC vs. CT	0.29 (0.41) NS	GG vs. GA	0.19 (0.40) NS
	AA vs. GG	− 1.06 (0.81) NS	CC vs. TT	0.11 (0.64) NS	CC vs. TT	1.21 (0.59) *P* = 0.040749	CC vs. TT	0.45 (0.58) NS	GG vs. AA	− 0.16 (0.94) NS
SBP (mmHg)	AA vs. AG	4.48 (1.49) *P* = 0.00279	CC vs. CT	0.35 (2.34) NS	CC vs. CT	− 0.84 (1.83) NS	CC vs. CT	0.29 (1.52) NS	GG vs. GA	2.48 (1.49) NS
	AA vs. GG	− 1.35 (3.00) NS	CC vs. TT	− 1.41 (2.42) NS	CC vs. TT	0.95 (2.21) NS	CC vs. TT	3.93 (2.16) NS	GG vs. AA	6.07 (3.49) NS
DBP (mmHg)	AA vs. AG	1.66 (0.96) NS	CC vs. CT	− 1.14 (1.50) NS	CC vs. CT	0.00 (1.17) NS	CC vs. CT	1.18 (0.97) NS	GG vs. GA	1.00 (0.95) NS
	AA vs. GG	− 1.75 (1.93) NS	CC vs. TT	− 1.98 (1.55) NS	CC vs. TT	0.83 (1.41) NS	CC vs. TT	3.20 (1.38) *P* = 0.021	GG vs. AA	3.91 (2.24) NS
Total cholesterol (mg/dL)	AA vs. AG	1.30 (3.10) NS	CC vs. CT	0.26 (4.81) NS	CC vs. CT	− 0.68 (3.75 NS)	CC vs. CT	0.28 (3.14) NS	GG vs. GA	10.44 (3.04) *P* = 0.000654
	AA vs. GG	2.65 (6.29) NS	CC vs. TT	3.36 (4.97 NS	CC vs. TT	− 9.39 (4.55) *P* = 0.0395	CC vs. TT	5.36 (4.50) NS	GG vs. AA	14.36 (7.18) *P* = 0.046152
LDL (mg/dL)	AA vs. AG	− 0.43 (2.77) NS	CC vs. CT	− 0.30 (4.30) NS	CC vs. CT	− 0.90 (3.35) NS	CC vs. CT	− 1.45 (2.81) NS	GG vs. GA	9.98 (2.72) *P* = 0.000264
	AA vs. GG	1.50 (5.61) NS	CC vs. TT	1.68 (4.44) NS	CC vs. TT	− 8.04 (4.06) *P* = 0.0484	CC vs. TT	3.36 (4.02) NS	GG vs. AA	10.48 (6.41) NS
HDL (mg/dL)	AA vs. AG	1.63 (0.94) NS	CC vs. CT	1.18 (1.47) NS	CC vs. CT	1.21 (1.15) NS	CC vs. CT	1.59 (0.96) NS	GG vs. GA	− 0.55 (0.94) NS
	AA vs. GG	1.89 (1.91) NS	CC vs. TT	2.10 (1.51) NS	CC vs. TT	1.28 (1.39) NS	CC vs. TT	1.08 (1.37) NS	GG vs. AA	0.23 (2.22) NS
Glucose (mg/dL)	AA vs. AG	− 3.54 (2.88) NS	CC vs. CT	− 0.32 (4.47) NS	CC vs. CT	− 5.03 (3.50) NS	CC vs. CT	0.071 (2.932) NS	GG vs. GA	− 1.43 (2.87) NS
	AA vs. GG	− 6.22 (5.85) NS	CC vs. TT	− 6.38 (4.62) NS	CC vs. TT	− 10.06 (4.24) *P* = 0.01805	CC vs. TT	− 2.83 (4.20) NS	GG vs. AA	− 4.25 (6.78) NS
Leptin (mg/dL)	AA vs. AG	0.85 (0.77) NS	CC vs. CT	− 0.56 (1.20) NS	CC vs. CT	1.67 (0.94) NS	CC vs. CT	0.42 (0.79) NS	GG vs. GA	− 0.76 (0.77) NS
	AA vs. GG	− 1.24 (1.57) NS	CC vs. TT	0.24 (1.24) NS	CC vs. TT	2.60 (1.14) *P* = 0.02253	CC vs. TT	0.34 (1.12) NS	GG vs. AA	− 0.11 (1.82) NS

HDL, high-density lipoprotein; LDL, low-density lipoprotein.

Table 3 shows the association of the genotypes with the prevalence of metabolic and cardiovascular diseases; the coefficients for the confounding variables and model *R*^2^ values are shown in S9–S13 Tables. The TT type of rs174570, which was the derived allele but dominant in Melanesian populations (Kusaghe and Munda Town), significantly reduced the risk of hypertension (odds ratio [confidence interval] = 0.90 [0.81–0.997] *P* = 0.045) compared with the CC type. The GA type of rs2722425 was significantly associated with high cholesterol (*P* = 0.014309) and high LDL (*P* = 0.002849). Other types that were significantly associated with health indicators (Table 1) were not related to the occurrence of diseases to a significant degree. No associations remained significant after Bonferroni’s adjustment.

**Table 3:** The effects of tropical-climate-related SNPs and root-crop-diet-related SNPs on the occurrence of diseases; logistic regression analyses, after adjustment for age, gender, and population differences (also see S9–S13 Tables).

	rs162036		rs174570		rs185819		rs2237892		rs2722425	
Overweight (BMI ≥25 kg/m^2^)	AA vs. AG	1.36 [0.90–2.06] NS	CC vs. CT	0.74 [0.38–1.41] NS	CC vs. CT	1.08 [0.66–1.77] NS	CC vs. CT	0.94 [0.62–1.43] NS	GG vs. GA	0.79 [0.52–1.19] NS
	AA vs. GG	0.43 [0.16–1.05] NS	CC vs. TT	0.78 [0.40–1.51] NS	CC vs. TT	1.73 [0.96–3.13] NS	CC vs. TT	1.40 [0.77–2.57] NS	GG vs. AA	0.41 [0.15–1.09] NS
Diabetes (serum glucose ≥110 mg/dL	AA vs. AG	0.99 [0.94–1.05] NS	CC vs. CT	1.01 [0.93–1.10] NS	CC vs. CT	0.98 [0.92–1.05] NS	CC vs. CT	0.97 [0.92–1.02] NS	GG vs. GA	0.99 [0.94–1.05] NS
	AA vs. GG	0.98 [0.88–1.08] NS	CC vs. TT	0.97 [0.89–1.05] NS	CC vs. TT	0.96 [0.89–1.04] NS	CC vs. TT	0.95 [0.88–1.03] NS	GG vs. AA	0.99 [0.87–1.12] NS
Hypertension (SBP ≥140 mmHg and/or DBP ≥90 mmHg)	AA vs. AG	1.06 [0.999–1.13] NS	CC vs. CT	0.94 [0.85–1.04] NS	CC vs. CT	0.95 [0.88–1.03] NS	CC vs. CT	1.05 [0.98–1.12] NS	GG vs. GA	1.02 [0.96–1.09] NS
	AA vs. GG	0.99 [0.87–1.12] NS	CC vs. TT	0.90 [0.81–0.997] *P* = 0.04512	CC vs. TT	1.01 [0.92–1.11] NS	CC vs. TT	1.05 [0.96–1.15] NS	GG vs. AA	1.12 [0.96–1.29] NS
High cholesterol (serum chorlesterol≥240 mg/dL)	AA vs. AG	0.99 [0.95–1.04] NS	CC vs. CT	1.00 [0.94–1.07] NS	CC vs. CT	1.04 [0.99–1.10] NS	CC vs. CT	0.99 [0.95–1.04] NS	GG vs. GA	1.05 [1.01–1.10] *P* = 0.014309
	AA vs. GG	1.00 [0.92–1.09] NS	CC vs. TT	1.03 [0.96–1.10] NS	CC vs. TT	0.99 [0.94–1.06] NS	CC vs. TT	1.05 [0.99–1.12] NS	GG vs. AA	1.08 [0.98–1.19] NS
High LDL (serum LDL ≥140 mg/dL)	AA vs. AG	0.99 [0.92–1.06] NS	CC vs. CT	1.04 [0.93–1.17] NS	CC vs. CT	0.97 [0.88–1.06] NS	CC vs. CT	0.99 [0.92–1.07] NS	GG vs. GA	1.12 [1.04–1.20] *P* = 0.002849
	AA vs. GG	1.00 [0.86–1.16] NS	CC vs. TT	1.05 [0.93–1.18] NS	CC vs. TT	0.93 [0.83–1.03] NS	CC vs. TT	1.05 [0.94–1.17] NS	GG vs. AA	1.07 [0.90–1.27] NS

BMI, body mass index; DBP, diastolic blood pressure; HDL, high-density lipoprotein; LDL, low-density lipoprotein; SBP, systolic blood pressure.

## Discussion

This study analyzed the effect of the SNPs, which were found to be either adaptive or maladaptive in regions with a high-temperature, humid environment, and a root-crop diet and as risk factors for the metabolic and the cardiovascular diseases in a recent GWAS study, on the populations of the Solomon Islands. These SNPs were chosen in replicating analyses and these findings were confirmed in previous GWAS studies, and thus many potential associations with the health variables and the occurrences of diseases were identified and confirmed.

The findings of the allele frequencies of rs174570 and rs185819 among Solomon Islands populations supported the results of a previous study that the derived alleles were adaptive for a high-temperature, humid environment [15]. Since rs185819 was associated with a high summer temperature rather than the temperature throughout the year, the high proportion of the T carriers of rs185819 in Ravaki Village is thought to be due to the fact that Kiribati (their original location) is located at a higher latitude than Melanesia.

The allele frequencies of rs162036 and the rs2237892 also supported previous findings that these were adaptive for a diet consisting largely of tuberous crops (a non-cereal diet). However, the allele frequency of rs2722425 in the Solomon Islands populations in this study and Melanesians in the HGDP differed from that noted in other populations. Since no data are available regarding the allele frequency of this SNP in other AN-speaking Pacific Islands populations, further studies will be necessary to discuss this finding in an appropriate context. However, since the derived allele was associated with an increased risk of cardiovascular disease in this study, this allele may indeed have functioned in selective pressure in this region. As an overall pattern, the allele frequencies of the NAN-speaking Melanesians (HGDP) were similar to those of the AN-speaking Melanesia (present study), regardless of difference in the eras of migration and settlement, suggesting that these genotypes were susceptible to the environment and subsistence in Melanesia.

Next we attempted to make a functional explanation of these SNPs. Because the *MTRR* plays a role in the dietary folate metabolism, it is reasonable that the derived allele (G type of rs162036) increased in the low folate diet populations. The folate metabolism plays an important role in the development of fetus and cell homeostasis and, as in previous studies [15, 43], we also showed in the present study that the AG type was associated with an elevated BMI. The proportion of the GG type was so small in the study population (N = 33(5.9%)) that this small sample size may have overlooked the effects of this genotype; especially influenced by a fact that only 2.4% were from this genotype in our samples of the Micronesian population, who were more likely to be obese (S1 Table) [7, 8, 10, 11].

Rs174570 is located in the intron region in *FADS2* and no other SNPs have been reported as in LD with this SNP. This gene has been known to regulate the unsaturation of fatty acids and thus is associated with cardiovascular risks. The hypertensive predisposition (e.g., polymorphisms in *AGT*) has also been known to be originally adaptive in tropical environments, but it is a risk in today’s modern lifestyle [9, 44, 45]. Our previous study found that the ancestral types (risk allele) of polymorphisms in the hypertension susceptibility *AGT* gene were prevalent in the populations of the Solomon Islands and its rs5049 polymorphism was associated with hypertension. The ancestral type of rs174570 also showed a positive association with hypertension and it is reasonable to think that selection pressure in such a tropical environment caused this allele frequency and hypertension risk in the Solomon Islands.

Rs185819 is the missense variation of *TNXB*. Tenascins are extracellular matrix glycoproteins and they play a role developing embryos and healing wounds. While some previous studies have reported rs185819 to be associated with height, we found it not to be associated with height, but instead with the BMI [16, 30]. This finding may therefore be interpreted as suggesting that this allele is related to body size in general. Its function in high-summer temperatures has yet to be clarified, but wounds can pose a survival risk in such environments through tropical ulcers and other source of infections and therefore it is natural to consider that this gene is related with selection in this environment. The derived allele of this SNP was also associated with decreased cholesterol and LDL levels, while it increased the BMI. This may be influenced by the fact that the Micronesian population, who were more obese, but had lower cholesterol and LDL than the urban Melanesian population, probably due to different cultures and lifestyles [7–11] and that the GG type was very prevalent in the Ravaki population (Fig 1, S1 Table).

Rs2237892 of *KCNQ1* was similarly associated with elevated blood pressure, as reported in a previous study [39]. This SNP is located in the intron region, but it is also known as in LD with rs2283228 and rs7480855, both of which are also in the intron, according to Regulome Database from HapMap 2 Project data[46]. Since the *KCNQ1* encodes a potassium channel protein in cardiac tissue, this gene is known to be associated with cardiovascular risks [39]. This gene is also known to affect the secretions of glucagon-like peptide (GLP)-1 and glucose-dependent insulinotropic peptide (GIP) from the gut, which are stimulated by dietary fiber, especially from cereals [47–51], so that the derived alleles were prevalent in the populations of the Solomon Islands who originally did not primarily depend on dietary cereals. Despite the fact that many previous studies have reported that rs2237892 is associated with diabetes, no such association was found in this study, thus suggesting this SNP might not function as a diabetes risk factor in populations that do not depend primarily on a cereal diet.

In addition, we found that rs2722425 had the strongest effects on health among the SNPs analyzed, through elevated cholesterol and LDL levels. This SNP is located in the intron region without any known linkage disequilibrium. Zinc finger is a small protein structural motif which stabilizes the fold and functions in DNA recognition, RNA packaging, transcriptional activation, the regulation of apoptosis, protein folding and assembly, and lipid binding, but not all functions have yet been revealed [52]. This study questioned the previous finding that this gene is associated with a tuber crop diet. The finding that the SNP in this gene is strongly associated with the metabolism may contribute to obtaining a better understanding of the function of the zinc finger proteins. Although the effect of the derived allele was significant for the GA type but not the AA type, the AA type also increased the LDL level (with a larger coefficient than the GA type) at a non-significant level. This non-significance was thought to be due to the fact that the proportion of AA type was very small in our study population. Interpretation of the association between genotypes and disease risks is complicated, since such genotypes were adaptive to the environment and subsistence in prehistoric times but now carry risks of disease under a modern Westernized lifestyle, according to the thrifty genotype hypothesis. For rs2722425, as shown in S13 Table, a high prevalence of high cholesterol and high LDL was observed among the derived allele carriers both in rural and urban Melanesian populations but not in the Micronesian population. We may therefore reasonably conclude that this risk existed before Westernization and may have functioned in selection pressure. If this was indeed the case, such selection might have happened in the Pacific environment, since the derived allele was seldom found in both AN-speaking (this study) and NAN-speaking (HGDP) populations.

Although further studies are necessary, the Solomon Islands populations in our study showed that the genetic polymorphisms associated with climate and diet have been influenced by unique selection pressure occurring in AN-speaking Pacific Islands populations. Identifying such genotypes may help mitigate the epidemic of non-communicable diseases in the Pacific Islands.

## Supporting information

S1 Table The allele frequencies of five SNPs in the populations analyzed in the Human Genome Diversity Project (HGDP) and in this study (also see Fig 1). (DOCX)

S2 Table The effects of the variant allele of rs162036 on health variables. (DOCX)

S3 Table The effects of the variant allele of rs174570 on health variables. (DOCX)

S4 Table The effects of the variant allele of rs185819 on health variables. (DOCX)

S5 Table The effects of the variant allele of rs2237892 on health variables. (DOCX)

S6 Table The effects of the variant allele of rs2722425 on health variables. (DOCX)

S7 Table The effects of the variant allele of 5 SNPs on body height, broken down by gender. (DOCX)

S8 Table The effects of the variant allele of 5 SNPs on serum leptin, broken down by gender. (DOCX)

S9 Table The effects of the variant allele of rs162036 on the occurrence of diseases. (DOCX)

S10 Table The effects of the variant allele of rs174570 on the occurrence of diseases. (DOCX)

S11 Table The effects of the variant allele of rs185819 on the occurrence of diseases. (DOCX)

S12 Table The effects of the variant allele of rs2237892 on the occurrence of diseases. (DOCX)

S13 Table The effects of the variant allele of rs2722425 on the occurrence of diseases. (DOCX)
