# Supplementary material for: Polymorphisms associated with a tropical climate and root crop diet induce susceptibility to metabolic and cardiovascular diseases in Solomon Islands
Source: PLoS One. 2017 Mar 2;12(3):e0172676. doi: 10.1371/journal.pone.0172676 (PMC5333831; doi:10.1371/journal.pone.0172676)
Supplement: S1 Table — (DOCX) [file pone.0172676.s001.docx]

S1 Table. The allele frequencies of five SNPs in the populations analyzed in the human genome diversity project (HGDP) and in this study (also see Figure 1).

|  | rs162036 | | | rs174570 | | | rs185819 | | | rs2237892 | | | rs2722425 | | |
| --- | --- | --- | --- | --- | --- | --- | --- | --- | --- | --- | --- | --- | --- | --- | --- |
| Population | AA | AG | GG | CC | TC | TT | CC | TC | TT | CC | TC | TT | GG | GA | AA |
| HGDP: East Asia | 161 | 64 | 9 | 72 | 107 | 55 | 114 | 85 | 35 | 93 | 110 | 31 | 133 | 85 | 16 |
| HGDP: Europe | 119 | 37 | 4 | 122 | 38 | 0 | 60 | 75 | 25 | 148 | 12 | 0 | 126 | 33 | 1 |
| HGDP: Melanesia | 17 | 17 | 2 | 5 | 20 | 11 | 5 | 18 | 13 | 18 | 14 | 4 | 27 | 9 | 0 |
| HGDP: Middle East | 105 | 35 | 6 | 122 | 20 | 4 | 75 | 58 | 12 | 133 | 13 | 0 | 105 | 35 | 6 |
| HGDP: North Africa | 14 | 12 | 4 | 23 | 7 | 0 | 4 | 17 | 9 | 24 | 6 | 0 | 21 | 9 | 0 |
| HGDP: Latin America | 25 | 33 | 50 | 0 | 9 | 99 | 76 | 30 | 2 | 23 | 51 | 34 | 47 | 33 | 28 |
| HGDP: South Asia | 146 | 48 | 3 | 140 | 51 | 6 | 79 | 101 | 17 | 173 | 24 | 0 | 140 | 49 | 8 |
| HGDP: Southeast Asia | 11 | 0 | 0 | 1 | 4 | 6 | 4 | 7 | 0 | 6 | 4 | 1 | 6 | 4 | 1 |
| HGDP: Subsaharan Africa | 58 | 43 | 20 | 121 | 0 | 0 | 39 | 59 | 23 | 85 | 35 | 1 | 40 | 55 | 26 |
| This Study: Munda | 107 | 67 | 9 | 17 | 87 | 79 | 54 | 99 | 30 | 56 | 93 | 34 | 121 | 56 | 6 |
| This Study: Paradise | 89 | 107 | 19 | 15 | 79 | 121 | 60 | 110 | 45 | 104 | 88 | 23 | 135 | 76 | 4 |
| This Study: Ravaki | 115 | 44 | 4 | 31 | 89 | 43 | 13 | 41 | 109 | 73 | 68 | 23 | 72 | 77 | 15 |

This study analyzed the Kusaghe (rural Melanesian, N = 183), Munda (urban Melanesian, N = 215), and Ravaki peoples (Micronesian, N = 163); Ravaki people were sampled from the Solomon Islands, and their original location is shown on the map. HGDP East Asia (N = 234) includes the following geographic origins: China, Japan, Siberia; HGDP Europe (N = 160): France, Italy, Italy (Bergamo), Orkney Islands, Russia, Russia (Caucasus); HGDP Melanesia (N = 36): Bougainville, Papua New Guinea, HGDP Middle East (N = 146): Israel (Carmel), Israel (Central), Israel (Negev); HGDP North Africa (N = 30): Algeria (Mzab); HGDP Latin America (N = 108): Brazil, Colombia, and Mexico; HGDP South Asia (N = 197): Pakistan; HGDP Southeast Asia (N = 11): Cambodia; and HGDP Sub-Saharan Africa (N = 121): Central African Republic, Democratic Republic of Congo, Kenya, Namibia, Nigeria, Senegal, and South Africa.
