# Supplementary material for: Polymorphisms associated with a tropical climate and root crop diet induce susceptibility to metabolic and cardiovascular diseases in Solomon Islands
Source: PLoS One. 2017 Mar 2;12(3):e0172676. doi: 10.1371/journal.pone.0172676 (PMC5333831; doi:10.1371/journal.pone.0172676)
Supplement: S2 Table — (DOCX) [file pone.0172676.s002.docx]

S2 Table. The effects of the variant allele of rs162036 on health variables

|  | Polymorphism | | Age | Sex  (Female = 0;  Male = 1) | Population difference  (Kusaghe = 0) | | Intercept | Model-adjusted *R^2^* |
| --- | --- | --- | --- | --- | --- | --- | --- | --- |
|  |  |  |  |  | Munda = 1 | Ravaki = 1 |  | Model *P* |
| Body height (cm) | AA vs. AG | -0.20 (0.51)  NS | -0.12 (0.02)  *P<*0.0001 | 9.97 (0.48)  *P<*0.0001 | 1.38 (0.57)  *P =* 0.015 | 5.77 (0.64)  *P<*0.0001 | 158.95 (0.83)  *P<*0.0001 | *R^2^*=0.53  *P<*0.0001 |
|  | AA vs. GG | -0.71 (1.04)  NS |  |  |  |  |  |  |
| Body weight (kg) | AA vs. AG | 2.00 (1.15)  NS | -0.046 (0.039)  NS | 2.45 (1.08)  *P =* 0.0232 | 5.34 (1.28)  *P<*0.0001 | 18.42 (1.44)  *P<*0.0001 | 60.75 (1.88)  *P<*0.0001 | *R^2^*=0.26  *P<*0.0001 |
|  | AA vs. GG | -3.06 (2.33)  NS |  |  |  |  |  |  |
| BMI (kg/m^2^) | AA vs. AG | 0.80 (0.40)  *P =* 0.04190 | 0.015 (0.013)  NS | -2.20 (0.37)  *P<*0.0001 | 1.66 (0.44)  *P =* 0.0002 | 5.05 (0.50)  *P<*0.0001 | 24.26 (0.65)  *P<*0.0001 | *R^2^*=0.22  *P<*0.0001 |
|  | AA vs. GG | -1.06 (0.81)  NS |  |  |  |  |  |  |
| SBP (mmHg) | AA vs. AG | 4.48 (1.49)  *P =* 0.00279 | 0.38 (0.05) *P<*0.0001 | 1.06 (1.39) NS | 4.72 (1.65) *P =* 0.00436 | -0.12 (1.87) NS | 102.82 (2.42) *P<*0.0001 | *R^2^*=0.15 *P<*0.0001 |
|  | AA vs. GG | -1.35 (3.00)  NS |  |  |  |  |  |  |
| DBP (mmHg) | AA vs. AG | 1.66 (0.96)  *P =* 0.0842 | 0.166 (0.032) *P<*0.0001 | -4.94 (0.90) *P<*0.0001 | 6.57 (1.06) *P<*0.0001 | 6.05 (1.21) *P<*0.0001 | 66.77 (1.56) *P<*0.0001 | *R^2^*=0.18 *P<*0.0001 |
|  | AA vs. GG | -1.75 (1.93)  NS |  |  |  |  |  |  |
| Total cholesterol (mg/dL) | AA vs. AG | 1.30 (3.10)  NS | 1.01 (0.11) *P<*0.0001 | -18.21 (2.91) *P<*0.0001 | -1.63 (3.44) NS | -10.83 (3.88) *P =* 0.00544 | 150.27 (5.06) *P<*0.0001 | *R^2^*=0.22 *P<*0.0001 |
|  | AA vs. GG | 2.65 (6.29)  NS |  |  |  |  |  |  |
| LDL (mg/dL) | AA vs. AG | -0.43 (2.77)  NS | 0.86 (0.09) *P<*0.0001 | -13.15 (2.60) *P<*0.0001 | 5.43 (3.07) *P =* 0.0777 | 2.69 (3.47) NS | 88.37 (4.52) *P<*0.0001 | *R^2^*=0.19 *P<*0.0001 |
|  | AA vs. GG | 1.50 (5.61)  NS |  |  |  |  |  |  |
| HDL (mg/dL) | AA vs. AG | 1.63 (0.94)  NS | -0.055 (0.03) NS | -6.37 (0.88) *P<*0.0001 | -6.77 (1.05) *P<*0.0001 | -10.97 (1.18) *P<*0.0001 | 55.28 (1.54) *P<*0.0001 | *R^2^*=0.25 *P<*0.0001 |
|  | AA vs. GG | 1.89 (1.91)  NS |  |  |  |  |  |  |
| Glucose (mg/dL) | AA vs. AG | -3.54 (2.88)  NS | 0.59 (0.10) *P<*0.0001 | -7.18 (2.71) *P =* 0.00822 | -5.13 (3.20) | 4.19 (3.61) | 78.31 (4.71) *P<*0.0001 | *R^2^*=0.071 *P<*0.0001 |
|  | AA vs. GG | -6.22 (5.85)  NS |  |  |  |  |  |  |
| Leptin (mg/dL) | AA vs. AG | 0.85 (0.77)  NS | 0.031 (0.026)  NS | -12.09 (0.72) *P<*0.0001 | 6.42 (0.86) *P<*0.0001 | 3.97 (0.97) *P<*0.0001 | 11.35 (1.26) *P<*0.0001 | *R^2^*=0.40 *P<*0.0001 |
|  | AA vs. GG | -1.24 (1.57)  NS |  |  |  |  |  |  |

BMI, body mass index; DBP, diastolic blood pressure; HDL, high-density lipoprotein; LDL, low-density lipoprotein; SBP, systolic blood pressure
