# Supplementary material for: Polymorphisms associated with a tropical climate and root crop diet induce susceptibility to metabolic and cardiovascular diseases in Solomon Islands
Source: PLoS One. 2017 Mar 2;12(3):e0172676. doi: 10.1371/journal.pone.0172676 (PMC5333831; doi:10.1371/journal.pone.0172676)
Supplement: S3 Table — (DOCX) [file pone.0172676.s003.docx]

S3 Table. The effects of the variant allele of rs174570 on health variables

|  | Polymorphism | | Age | Sex  (Female = 0;  Male = 1) | Population difference | | Intercept | Model-adjusted *R^2^* |
| --- | --- | --- | --- | --- | --- | --- | --- | --- |
|  |  |  |  |  | Munda = 1 | Ravaki = 1 |  | Model *P* |
| Body height (cm) | CC vs. CT | 1.09 (0.79)  NS | -0.12 (0.02) *P<*0.0001 | 10.01 (0.48) *P<*0.0001 | 1.46 (0.56)  *P =* 0.010 | 6.04 (0.65) *P<*0.0001 | 157.71 (1.06) *P<*0.0001 | *R^2^*=0.53 *P<*0.0001 |
|  | CC vs. TT | 1.11 (0.82)  NS |  |  |  |  |  |  |
| Body weight (kg) | CC vs. CT | 0.54 (1.80)  NS | -0.046 (0.039)  NS | 2.41 (1.08) *P =* 0.0265 | 5.32 (1.28) *P<*0.0001 | 18.49 (1.48) *P<*0.0001 | 60.59 (2.41) *P<*0.0001 | *R^2^*=0.25 *P<*0.0001 |
|  | CC vs. TT | 1.28 (1.86)  NS |  |  |  |  |  |  |
| BMI (kg/m^2^) | CC vs. CT | -0.13 (0.62)  NS | 0.015 (0.014)  NS | -2.23 (0.37) *P<*0.0001 | 1.63 (0.44) *P =* 0.000251 | 5.00 (0.51) *P<*0.0001 | 24.58 (0.83) *P<*0.0001 | *R^2^*=0.21 *P<*0.0001 |
|  | CC vs. TT | 0.11 (0.64)  NS |  |  |  |  |  |  |
| SBP (mmHg) | CC vs. CT | 0.35 (2.34)  NS | 0.37 (0.05) *P<*0.0001 | 0.80 (1.40)  NS | 4.00 (1.66) *P =* 0.0161 | -1.71 (1.93)  NS | 105.92 (3.12) *P<*0.0001 | *R^2^* = 0.14 *P<*0.0001 |
|  | CC vs. TT | -1.41 (2.42)  NS |  |  |  |  |  |  |
| DBP (mmHg) | CC vs. CT | -1.14 (1.50)  NS | 0.16 (0.03) *P<*0.0001 | -5.02 (0.90) *P<*0.0001 | 6.31 (1.06) *P<*0.0001 | 5.26 (1.23) *P<*0.0001 | 69.12 (2.00) *P<*0.0001 | *R^2^*=0.18 *P<*0.0001 |
|  | CC vs. TT | -1.98 (1.55)  NS |  |  |  |  |  |  |
| Total cholesterol (mg/dL) | CC vs. CT | 0.26 (4.81)  NS | 1.01 (0.11) *P<*0.0001 | -18.26 (2.90) *P<*0.0001 | -1.48 (3.43)  NS | -10.16 (3.95) *P =* 0.0105 | 149.19 (6.46) *P<*0.0001 | *R^2^*=0.22 *P<*0.0001 |
|  | CC vs. TT | 3.36 (4.97  NS |  |  |  |  |  |  |
| LDL (mg/dL) | CC vs. CT | -0.30 (4.30)  NS | 0.86 (0.09) *P<*0.0001 | -13.11 (2.59) *P<*0.0001 | 5.68 (3.06)  NS | 3.36 (3.53)  NS | 87.40 (5.77) *P<*0.0001 | *R^2^*=0.19 *P<*0.0001 |
|  | CC vs. TT | 1.68 (4.44)  NS |  |  |  |  |  |  |
| HDL (mg/dL) | CC vs. CT | 1.18 (1.47)  NS | -0.055 (0.032) *P =* 0.084 | -6.47 (0.88) *P<*0.0001 | -6.90 (1.04) *P<*0.0001 | -10.95 (1.20) *P<*0.0001 | 54.71 (1.97) *P<*0.0001 | *R^2^*=0.25 *P<*0.0001 |
|  | CC vs. TT | 2.10 (1.51)  NS |  |  |  |  |  |  |
| Glucose (mg/dL) | CC vs. CT | -0.32 (4.47)  NS | 0.60 (0.10) *P<*0.0001 | -7.00 (2.69)  *P =* 0.00958 | -5.26 (3.18)  NS | 3.19 (3.67)  NS | 79.57 (6.00) *P<*0.0001 | *R^2^*=0.077 *P<*0.0001 |
|  | CC vs. TT | -6.38 (4.62)  NS |  |  |  |  |  |  |
| Leptin (mg/dL) | CC vs. CT | -0.56 (1.20)  NS | 0.031 (0.026)  NS | -12.11 (0.72) *P<*0.0001 | 6.45 (0.86) *P<*0.0001 | 4.04 (0.99) *P<*0.0001 | 11.76 (1.61) *P<*0.0001 | *R^2^*=0.40 *P<*0.0001 |
|  | CC vs. TT | 0.24 (1.24)  NS |  |  |  |  |  |  |

BMI, body mass index; DBP, diastolic blood pressure; HDL, high-density lipoprotein; LDL, low-density lipoprotein; SBP, systolic blood pressure
