# Supplementary material for: Polymorphisms associated with a tropical climate and root crop diet induce susceptibility to metabolic and cardiovascular diseases in Solomon Islands
Source: PLoS One. 2017 Mar 2;12(3):e0172676. doi: 10.1371/journal.pone.0172676 (PMC5333831; doi:10.1371/journal.pone.0172676)
Supplement: S4 Table — (DOCX) [file pone.0172676.s004.docx]

S4 Table. The effects of the variant allele of rs185819 on health variables

|  | Polymorphism | | Age | Sex  (Female = 0;  Male = 1) | Population difference | | Intercept | Model-adjusted *R^2^* |
| --- | --- | --- | --- | --- | --- | --- | --- | --- |
|  |  |  |  |  | Munda = 1 | Ravaki = 1 |  | Model *P* |
| Body height (cm) | CC vs. CT | 0.77 (0.62)  NS | -0.12 (0.02) *P<*0.0001 | 10.00 (0.48) *P<*0.0001 | 1.41 (0.56)  *P =* 0.0126 | 6.03 (0.71) *P<*0.0001 | 158.28 (0.90) *P<*0.0001 | *R^2^* = 0.53 *P<*0.0001 |
|  | CC vs. TT | 0.11 (0.75)  NS |  |  |  |  |  |  |
| Body weight (kg) | CC vs. CT | 1.59 (1.40)  NS | -0.047 (0.039) | 2.58 (1.08)  *P =* 0.0178 | 5.30 (1.27) *P<*0.0001 | 16.92 (1.61) *P<*0.0001 | 60.00 (2.04) *P<*0.0001 | *R^2^* = 0.25 *P<*0.0001 |
|  | CC vs. TT | 2.97 (1.70)  *P =* 0.0810 |  |  |  |  |  |  |
| BMI (kg/m^2^) | CC vs. CT | 0.38 (0.48)  NS | 0.014 (0.014) | -2.16 (0.37) *P<*0.0001 | 1.65 (0.44)  *P =* 0.000185 | 4.36 (0.56) *P<*0.0001 | 24.58 (0.83) *P<*0.0001 | *R^2^*=0.21 *P<*0.0001 |
|  | CC vs. TT | 1.21 (0.59)  *P =* 0.040749 |  |  |  |  |  |  |
| SBP (mmHg) | CC vs. CT | -0.84 (1.83)  NS | 0.37 (0.05) *P<*0.0001 | 0.88 (1.41) | 4.30 (1.65)  *P =* 0.00948 | -1.90 (2.10) | 105.53 (2.65) *P<*0.0001 | *R^2^* = 0.14 *P<*0.0001 |
|  | CC vs. TT | 0.95 (2.21)  NS |  |  |  |  |  |  |
| DBP (mmHg) | CC vs. CT | 0.00 (1.17)  NS | 0.16 (0.03) *P<*0.0001 | -4.95 (0.90) *P<*0.0001 | 6.47 (1.06) *P<*0.0001 | 5.29 (1.34) *P<*0.0001 | 67.36 (1.70) *P<*0.0001 | *R^2^* = 0.18 *P<*0.0001 |
|  | CC vs. TT | 0.83 (1.41)  NS |  |  |  |  |  |  |
| Total cholesterol (mg/dL) | CC vs. CT | -0.68 (3.75  NS) | 1.02 (0.20) *P<*0.0001 | -18.95 (2.90) *P<*0.0001 | -2.37 (3.40) | -6.16 (4.31) | 153.54 (5.45) *P<*0.0001 | *R^2^* = 0.23 *P<*0.0001 |
|  | CC vs. TT | -9.39 (4.55)  *P =* 0.0395 |  |  |  |  |  |  |
| LDL (mg/dL) | CC vs. CT | -0.90 (3.35)  NS | 0.87 (0.09) *P<*0.0001 | -13.67 (2.59) *P<*0.0001 | 5.04 (3.03)  NS | 7.02 (3.85)  NS | 90.41 (4.87) *P<*0.0001 | *R^2^* = 0.20 *P<*0.0001 |
|  | CC vs. TT | -8.04 (4.06)  *P =* 0.0484 |  |  |  |  |  |  |
| HDL (mg/dL) | CC vs. CT | 1.21 (1.15)  NS | -0.055 (0.032) NS | -6.42 (0.89) *P<*0.0001 | -7.03 (1.04) *P<*0.0001 | -11.84 (1.32) *P<*0.0001 | 55.41 (1.67) *P<*0.0001 | *R^2^* = 0.24 *P<*0.0001 |
|  | CC vs. TT | 1.28 (1.39)  NS |  |  |  |  |  |  |
| Glucose (mg/dL) | CC vs. CT | -5.03 (3.50)  NS | 0.60 (0.10) *P<*0.0001 | -7.51 (2.70 *P =* 0.00562 | -4.78 (3.16) | 9.64 (4.02)  *P =* 0.01687 | 80.68 (5.08) *P<*0.0001 | *R^2^* = 0.08 *P<*0.0001 |
|  | CC vs. TT | -10.06 (4.24)  *P =* 0.01805 |  |  |  |  |  |  |
| Leptin (mg/dL) | CC vs. CT | 1.67 (0.94)  NS | 0.032 (0.026)  NS | -11.95 (0.72) *P<*0.0001 | 6.42 (0.85) *P<*0.0001 | 2.87 (1.08)  *P =* 0.0079 | 10.18 (1.36) *P<*0.0001 | *R^2^* = 0.41 *P<*0.0001 |
|  | CC vs. TT | 2.60 (1.14)  *P =* 0.02253 |  |  |  |  |  |  |

BMI, body mass index; DBP, diastolic blood pressure; HDL, high-density lipoprotein; LDL, low-density lipoprotein; SBP, systolic blood pressure
