# Supplementary material for: Polymorphisms associated with a tropical climate and root crop diet induce susceptibility to metabolic and cardiovascular diseases in Solomon Islands
Source: PLoS One. 2017 Mar 2;12(3):e0172676. doi: 10.1371/journal.pone.0172676 (PMC5333831; doi:10.1371/journal.pone.0172676)
Supplement: S5 Table — (DOCX) [file pone.0172676.s005.docx]

S5 Table. The effects of the variant allele of rs2237892 on health variables

|  | Polymorphism | | Age | Sex  (Female = 0;  Male = 1) | Population difference | | Intercept | Model-adjusted *R^2^* |
| --- | --- | --- | --- | --- | --- | --- | --- | --- |
|  |  |  |  |  | Munda = 1 | Ravaki = 1 |  | Model *P* |
| Body height (cm) | CC vs. CT | 0.75 (0.52)  NS | -0.12 (0.02) *P<*0.0001 | 9.96 (0.48) *P<*0.0001 | 1.22 (0.57)  *P =* 0.0322 | 5.78 (0.62) *P<*0.0001 | 158.22 (0.81) *P<*0.0001 | *R^2^*=0.53 *P<*0.0001 |
|  | CC vs. TT | 1.62 (0.74)  *P =* 0.0286 |  |  |  |  |  |  |
| Body weight (kg) | CC vs. CT | 1.54 (1.17)  NS | -0.044 (0.039)  NS | 2.36 (1.08)  *P =* 0.029393 | 4.82 (1.28) *P<*0.0001 | 17.97 (1.40) *P<*0.0001 | 60.52 (1.83) *P<*0.0001 | *R^2^*=0.25 *P<*0.0001 |
|  | CC vs. TT | 2.69 (1.68)  NS |  |  |  |  |  |  |
| BMI (kg/m^2^) | CC vs. CT | 0.29 (0.41)  NS | 0.016 (0.014)  NS | -2.24 (0.37) *P<*0.0001 | 1.53 (0.46) *P<*0.0001 | 4.90 (0.49) *P<*0.0001 | 24.42 (0.63) *P<*0.0001 | *R^2^*=0.21 *P<*0.0001 |
|  | CC vs. TT | 0.45 (0.58)  NS |  |  |  |  |  |  |
| SBP (mmHg) | CC vs. CT | 0.29 (1.52)  NS | 0.38 (2.16) *P<*0.0001 | 0.69 (1.40)  NS | 3.81 (1.66)  *P =* 0.0226 | -1.29 (1.83) | 104.49 (2.36) *P<*0.0001 | *R^2^*=0.14, *P<*0.0001 |
|  | CC vs. TT | 3.93 (2.16)  NS |  |  |  |  |  |  |
| DBP (mmHg) | CC vs. CT | 1.18 (0.97)  NS | 0.17 (0.03) *P<*0.0001 | -5.03 (0.90) *P<*0.0001 | 6.02 (1.06) *P<*0.0001 | 5.52 (1.17) *P<*0.0001 | 66.53 (1.51) *P<*0.0001 | *R^2^*=0.18 *P<*0.0001 |
|  | CC vs. TT | 3.20 (1.38)  *P =* 0.0210 |  |  |  |  |  |  |
| Total cholesterol (mg/dL) | CC vs. CT | 0.28 (3.14)  NS | 1.01 (0.11) *P<*0.0001 | -18.67 (2.90) *P<*0.0001 | -2.41 (3.45)  NS | -11.23 (3.76) *P =* 0.00297 | 150.48 (4.91) *P<*0.0001 | *R^2^*=0.22 *P<*0.0001 |
|  | CC vs. TT | 5.36 (4.50)  NS |  |  |  |  |  |  |
| LDL (mg/dL) | CC vs. CT | -1.45 (2.81)  NS | 0.86 (0.09) *P<*0.0001 | -13.48 (2.59) *P<*0.0001 | 5.25 (3.08)  NS | 2.92 (3.36)  NS | 88.48 (4.38) *P<*0.0001 | *R^2^*=0.19 *P<*0.0001 |
|  | CC vs. TT | 3.36 (4.02)  NS |  |  |  |  |  |  |
| HDL (mg/dL) | CC vs. CT | 1.59 (0.96)  NS | -0.06 (0.03)  NS | -6.49 (0.88) *P<*0.0001 | -7.28 (1.05) *P<*0.0001 | -11.52 (1.14) *P<*0.0001 | 55.62 (1.49) *P<*0.0001 | *R^2^*=0.25 *P<*0.0001 |
|  | CC vs. TT | 1.08 (1.37)  NS |  |  |  |  |  |  |
| Glucose (mg/dL) | CC vs. CT | 0.071 (2.932)  NS | 0.59 (0.10) *P<*0.0001 | -6.70 (2.70)  *P =* 0.0135 | -4.21 (3.22)  NS | 5.43 (3.51)  NS | 76.03 (4.58) *P<*0.0001 | *R^2^*= 0.07 *P<*0.0001 |
|  | CC vs. TT | -2.83 (4.20)  NS |  |  |  |  |  |  |
| Leptin (mg/dL) | CC vs. CT | 0.42 (0.79)  NS | 0.03 (0.03)  NS | -12.11 (0.72) *P<*0.0001 | 6.29 (0.86) *P<*0.0001 | 3.80 (0.94) *P<*0.0001 | 11.47 (1.23) *P<*0.0001 | *R^2^*=0.40 *P<*0.0001 |
|  | CC vs. TT | 0.34 (1.12)  NS |  |  |  |  |  |  |

BMI, body mass index; DBP, diastolic blood pressure; HDL, high-density lipoprotein; LDL, low-density lipoprotein; SBP, systolic blood pressure
