# Supplementary material for: Polymorphisms associated with a tropical climate and root crop diet induce susceptibility to metabolic and cardiovascular diseases in Solomon Islands
Source: PLoS One. 2017 Mar 2;12(3):e0172676. doi: 10.1371/journal.pone.0172676 (PMC5333831; doi:10.1371/journal.pone.0172676)
Supplement: S6 Table — (DOCX) [file pone.0172676.s006.docx]

S6 Table. The effects of the variant allele of rs2722425 on health variables

|  | Polymorphism | | Age | Sex  (Female = 0;  Male = 1) | Population difference | | Intercept | Model-adjusted *R^2^* |
| --- | --- | --- | --- | --- | --- | --- | --- | --- |
|  |  |  |  |  | Munda = 1 | Ravaki = 1 |  | Model *P* |
| Body height (cm) | GG vs. GA | -0.43 (0.51)  NS | -0.12 (0.02) *P<*0.0001 | 10.02 (0.48)  *P<*0.0001 | 1.42 (0.56)  *P =* 0.012 | 5.93 (0.64)  *P<*0.0001 | 158.88 (0.78)  *P<*0.0001 | *R^2^*=0.53  *P<*0.0001 |
|  | GG vs. AA | -0.37 (1.20)  NS |  |  |  |  |  |  |
| Body weight (kg) | GG vs. GA | 0.23 (1.15)  NS | -0.046 (0.039)  NS | 2.43 (1.08)  *P =* 0.0251 | 5.23 (1.27)  *P<*0.0001 | 18.10 (1.44)  *P<*0.0001 | 61.42 (1.77)  *P<*0.0001 | *R^2^*=0.25  *P<*0.0001 |
|  | GG vs. AA | -0.63 (2.71)  NS |  |  |  |  |  |  |
| BMI (kg/m^2^) | GG vs. GA | 0.19 (0.40)  NS | 0.02 (0.01)  NS | -2.22 (0.37)  *P<*0.0001 | 1.61 (0.44)  *P =* 0.000268 | 4.90 (0.50)  *P<*0.0001 | 24.53 (0.61)  *P<*0.0001 | *R^2^*=0.21  *P<*0.0001 |
|  | GG vs. AA | -0.16 (0.94)  NS |  |  |  |  |  |  |
| SBP (mmHg) | GG vs. GA | 2.48 (1.49)  NS | 0.36 (0.05)  *P<*0.0001 | 0.65 (1.40)  NS | 4.29 (1.64)  *P =* 0.0092 | -2.07 (1.87)  NS | 104.53 (2.28)  *P<*0.0001 | *R^2^*=0.14  *P<*0.0001 |
|  | GG vs. AA | 6.07 (3.49)  NS |  |  |  |  |  |  |
| DBP (mmHg) | GG vs. GA | 1.00 (0.95)  NS | 0.16 (0.03)  *P<*0.0001 | -5.08 (0.90)  *P<*0.0001 | 6.44 (1.05)  *P<*0.0001 | 5.13 (1.20)  *P<*0.0001 | 67.25 (1.46)  *P<*0.0001 | *R^2^*=0.18  *P<*0.0001 |
|  | GG vs. AA | 3.91 (2.24)  NS |  |  |  |  |  |  |
| Total cholesterol (mg/dL) | GG vs. GA | 10.44 (3.04)  *P =* 0.000654 | 0.98 (0.10)  *P<*0.0001 | -18.90 (2.87)  *P<*0.0001 | -1.45 (3.36)  NS | -13.90 (3.81)  *P =* 0.000295 | 148.39 (4.68)  *P<*0.0001 | *R^2^*=0.24  *P<*0.0001 |
|  | GG vs. AA | 14.36 (7.18)  *P =* 0.046152 |  |  |  |  |  |  |
| LDL (mg/dL) | GG vs. GA | 9.98 (2.72)  *P =* 0.000264 | 0.84 (0.09) *P<*0.0001 | -13.59 (2.56)  *P<*0.0001 | 5.88 (3.00)  NS | 0.58 (3.40)  NS | 85.46 (4.18)  *P<*0.0001 | *R^2^*=0.21  *P<*0.0001 |
|  | GG vs. AA | 10.48 (6.41)  NS |  |  |  |  |  |  |
| HDL (mg/dL) | GG vs. GA | -0.55 (0.94)  NS | -0.056 (0.032)  NS | -6.52 (0.88)  *P<*0.0001 | -7.07 (1.04)  *P<*0.0001 | -11.40 (1.18)  *P<*0.0001 | 56.59 (1.44)  *P<*0.0001 | *R^2^*=0.24  *P<*0.0001 |
|  | GG vs. AA | 0.23 (2.22)  NS |  |  |  |  |  |  |
| Glucose (mg/dL) | GG vs. GA | -1.43 (2.87)  NS | 0.60 (0.10)  *P<*0.0001 | -6.68 (2.71)  *P =* 0.0139 | -4.51 (3.18)  NS | 5.96 (3.60)  NS | 75.98 (4.42)  *P<*0.0001 | *R^2^*=0.069  *P<*0.0001 |
|  | GG vs. AA | -4.25 (6.78)  NS |  |  |  |  |  |  |
| Leptin (mg/dL) | GG vs. GA | -0.76 (0.77)  NS | 0.032 (0.026)  NS | -12.11 (0.72)  *P<*0.0001 | 6.32 (0.85)  *P<*0.0001 | 3.94 (0.96)  *P<*0.0001 | 11.93 (1.18)  *P<*0.0001 | *R^2^*=0.40  *P<*0.0001 |
|  | GG vs. AA | -0.11 (1.82)  NS |  |  |  |  |  |  |

BMI, body mass index; DBP, diastolic blood pressure; HDL, high-density lipoprotein; LDL, low-density lipoprotein; SBP, systolic blood pressure
