# Supplementary material for: Polymorphisms associated with a tropical climate and root crop diet induce susceptibility to metabolic and cardiovascular diseases in Solomon Islands
Source: PLoS One. 2017 Mar 2;12(3):e0172676. doi: 10.1371/journal.pone.0172676 (PMC5333831; doi:10.1371/journal.pone.0172676)
Supplement: S7 Table — (DOCX) [file pone.0172676.s007.docx]

S7 Table. The effects of the variant allele of 5 SNPs on body height, broken down by gender

|  | Polymorphism | | Age | Population difference | | Intercept | Model  -adjusted  *R^2^* |
| --- | --- | --- | --- | --- | --- | --- | --- |
|  |  |  |  | Munda = 1 | Ravaki = 1 |  | Model *P* |
| rs162036 |  |  |  |  |  |  |  |
| Male | AA vs. AG | -0.95 (0.78)  NS | -0.10  (0.03)  *P* <0.0001 | 2.30 (0.89)  *P* = 0.00995 | 6.67 (0.98)  *P* <0.0001 | 167.83 (1.18)  *P* <0.0001 | 0.2124  *P* <0.0001 |
|  | AA vs. GG | 0.39 (1.71)  NS |  |  |  |  |  |
| Female | AA vs. AG | 0.52 (0.66)  NS | -0.15  (0.02)  *P* <0.0001 | 0.59 (0.72)  NS | 4.95 (0.83)  *P* <0.0001 | 160.28 (1.08)  *P* <0.0001 | 0.269  *P* <0.0001 |
|  | AA vs. GG | -1.14 (1.26)  NS |  |  |  |  |  |
| rs174570 |  |  |  |  |  |  |  |
| Male | CC vs. CT | 2.35 (1.17)  *P* = 0.046359 | -0.09  (0.03)  *P* = 0.000238 | 2.37 (0.87)  *P* = 0.004899 | 7.32 (0.98)  *P* <0.0001 | 164.86 (1.58)  *P* <0.0001 | 0.2222  *P* <0.0001 |
|  | CC vs. TT | 2.60 (1.22)  *P* = 0.03381 |  |  |  |  |  |
| Female | CC vs. CT | 0.03 (1.06)  NS | -0.15  (0.02)  *P* <0.0001 | 0.49 (0.72)  NS | 4.76 (0.85)  *P* <0.0001 | 160.55 (1.36)  *P* <0.0001 | 0.2638  *P* <0.0001 |
|  | CC vs. TT | -0.16 (1.09)  NS |  |  |  |  |  |
| rs185819 |  |  |  |  |  |  |  |
| Male | CC vs. CT | 1.33 (0.91)  NS | -0.10  (0.03)  *P* = 0.00015 | 2.46 (0.88)  *P* = 0.0055 | 6.95 (1.08)  *P* <0.0001 | 166.50 (1.28)  *P* <0.0001 | 0.2146  *P* <0.0001 |
|  | CC vs. TT | 0.46 (1.14)  NS |  |  |  |  |  |
| Female | CC vs. CT | 0.42 (0.85)  NS | -0.15  (0.02)  *P* <0.0001 | 0.46 (0.72)  NS | 4.97 (0.94)  *P* <0.0001 | 160.22 (1.18)  *P* <0.0001 | 0.2648  *P* <0.0001 |
|  | CC vs. TT | -0.005 (0.993)  NS |  |  |  |  |  |
| rs2237892 |  |  |  |  |  |  |  |
| Male | CC vs. CT | 1.46 (0.80)  NS | -0.096  (0.025)  *P* = 0.000187 | 2.02 (0.88)  *P* = 0.023228 | 6.70 (0.94)  *P* <0.0001 | 166.50 (1.17)  *P* <0.0001 | 0.2197  *P* <0.0001 |
|  | CC vs. TT | 1.61 (1.08)  NS |  |  |  |  |  |
| Female | CC vs. CT | 0.12 (0.66)  NS | -0.15  (0.02)  *P* <0.0001 | 0.41 (0.72)  NS | 4.70 (0.80)  *P* <0.0001 | 160.19 (1.05)  *P* <0.0001 | 0.2719  *P* <0.0001 |
|  | CC vs. TT | 1.73 (1.01)  NS |  |  |  |  |  |
| rs2722425 |  |  |  |  |  |  |  |
| Male | GG vs. GA | -0.38 (0.79)  NS | -0.10  (0.03)  *P* = 0.000155 | 2.36 (0.87)  *P* = 0.006980 | 7.09 (0.99)  *P* <0.0001 | 167.48 (1.12)  *P* <0.0001 | 0.2098  *P* <0.0001 |
|  | GG vs. AA | -1.47 (1.58)  NS |  |  |  |  |  |
| Female | GG vs. GA | -0.53 (0.65)  NS | -0.15  (0.02)  *P* <0.0001 | 0.46 (0.72)  NS | 4.74 (0.82)  *P* <0.0001 | 160.60 (1.02)  *P* <0.0001 | 0.2671  *P* <0.0001 |
|  | GG vs. AA | 1.47 (1.95)  NS |  |  |  |  |  |
