# Supplementary material for: Polymorphisms associated with a tropical climate and root crop diet induce susceptibility to metabolic and cardiovascular diseases in Solomon Islands
Source: PLoS One. 2017 Mar 2;12(3):e0172676. doi: 10.1371/journal.pone.0172676 (PMC5333831; doi:10.1371/journal.pone.0172676)
Supplement: S8 Table — (DOCX) [file pone.0172676.s008.docx]

S8 Table. The effects of the variant allele of 5 SNPs on serum leptin, broken down by gender

|  | Polymorphism | | Age | Population difference | | Intercept | Model  -adjusted  *R^2^* |
| --- | --- | --- | --- | --- | --- | --- | --- |
|  |  |  |  | Munda = 1 | Ravaki = 1 |  | Model *P* |
| rs162036 |  |  |  |  |  |  |  |
| Male | AA vs. AG | 0.41 (0.49)  NS | 0.066  (0.016)  *P* <0.0001 | 3.47 (0.55)  *P* <0.0001 | 1.95 (0.61)  *P* = 0.0016 | -0.55 (0.74)  NS | 0.2296  *P* <0.0001 |
|  | AA vs. GG | 0.35 (1.07)  NS |  |  |  |  |  |
| Female | AA vs. AG | 1.28 (1.42)  NS | 0.003  (0.051)  NS | 9.20 (1.55)  *P* <0.0001 | 5.97 (1.79)  *P* = 0.0009 | 10.91 (2.33)  *P* <0.0001 | 0.1161  *P* <0.0001 |
|  | AA vs. GG | -3.09 (2.71)  NS |  |  |  |  |  |
| rs174570 |  |  |  |  |  |  |  |
| Male | CC vs. CT | -1.39 (0.73)  NS | 0.062  (0.016)  *P* = 0.000111 | 3.32 (0.54)  *P* <0.0001 | 1.48 (0.61)  *P* = 0.016185 | 1.27 (0.98)  NS | 0.2427  *P* <0.0001 |
|  | CC vs. TT | -1.68 (0.76)  *P* = 0.027751 |  |  |  |  |  |
| Female | CC vs. CT | 0.34 (2.28)  NS | 0.004  (0.051)  NS | 9.21 (1.55)  *P* <0.0001 | 6.40 (1.83)  *P* = 0.000536 | 10.03 (2.91)  *P* = 0.000673 | 0.1126  *P* <0.0001 |
|  | CC vs. TT | 2.13 (2.35)  NS |  |  |  |  |  |
| rs185819 |  |  |  |  |  |  |  |
| Male | CC vs. CT | 0.26 (0.57)  NS | 0.066  (0.016)  *P* <0.0001 | 3.40 (0.55)  *P* <0.0001 | 1.98 (0.67) *P* = 0.00366 | -0.45 (0.80)  NS | 0.2289  *P* <0.0001 |
|  | CC vs. TT | -0.13 (0.72) NS |  |  |  |  |  |
| Female | CC vs. CT | 2.81 (1.82)  NS | 0.003  (0.051)  NS | 8.84 (1.55)  *P* <0.0001 | 3.70 (2.00)  NS | 8.93 (2.52)  *P* = 0.000458 | 0.1242  *P* <0.0001 |
|  | CC vs. TT | 4.83 (2.12)  *P* = 0.023429 |  |  |  |  |  |
| rs2237892 |  |  |  |  |  |  |  |
| Male | CC vs. CT | 0.15 (0.50)  NS | 0.067  (0.016)  *P* <0.0001 | 3.28 (0.55)  *P* <0.0001 | 1.82 (0.59)  *P* = 0.00223 | -0.52 (0.73)  NS | 0.231  *P* <0.0001 |
|  | CC vs. TT | 0.73 (0.68)  NS |  |  |  |  |  |
| Female | CC vs. CT | 0.83 (1.43)  NS | 0.003  (0.051)  NS | 8.92 (1.56)  *P* <0.0001 | 5.65 (1.74)  *P* = 0.00133 | 11.00 (2.27)  *P* <0.0001 | 0.1075  *P* <0.0001 |
|  | CC vs. TT | 0.24 (2.18)  NS |  |  |  |  |  |
| rs2722425 |  |  |  |  |  |  |  |
| Male | GG vs. GA | -0.28 (0.50)  NS | 0.065  (0.016)  *P* <0.0001 | 3.39 (0.54)  *P* <0.0001 | 1.92 (0.62)  *P* = 0.0021 | -0.22 (0.70)  NS | 0.2283  *P* <0.0001 |
|  | GG vs. AA | -0.22 (0.98)  NS |  |  |  |  |  |
| Female | GG vs. GA | -1.01 (1.39)  NS | 0.0069  (0.051)  NS | 8.93 (1.55)  *P* <0.0001 | 5.68 (1.76)  *P* = 0.00138 | 11.62 (2.20)  *P* <0.0001 | 0.1083  *P* <0.0001 |
|  | GG vs. AA | 0.60 (4.21)  NS |  |  |  |  |  |
