# Supplementary material for: Polymorphisms associated with a tropical climate and root crop diet induce susceptibility to metabolic and cardiovascular diseases in Solomon Islands
Source: PLoS One. 2017 Mar 2;12(3):e0172676. doi: 10.1371/journal.pone.0172676 (PMC5333831; doi:10.1371/journal.pone.0172676)
Supplement: S9 Table — (DOCX) [file pone.0172676.s009.docx]

S9 Table. The effects of the variant allele of rs162036 on the occurrence of diseases

|  | Polymorphism | | Age | Sex  (Female = 0;  Male = 1) | Population difference  (Kusaghe = 0) | | Intercept | Nagelkerke *R^2^* |
| --- | --- | --- | --- | --- | --- | --- | --- | --- |
|  |  |  |  |  | Munda = 1 | Ravaki = 1 |  |  |
| Overweight (BMI ≥ 25 kg/m^2^) | AA vs. AG | 1.36 [0.90-2.06]  NS | 1.01 [1.00-1.03]  *P =* 0.039505 | 0.32 [0.21-0.47]  *P<*0.0001 | 2.26 [1.45-3.55]  *P =* 0.0004 | 8.67 [5.03-15.39]  *P<*0.0001 | 0.38  [0.19-0.74]  *P =* 0.004908 | 0.2483189 |
|  | AA vs. GG | 0.43 [0.16-1.05]  NS |  |  |  |  |  |  |
| Diabetes (serum glucose ≥110 mg/dL) | AA vs. AG | 0.99 [0.94-1.05]  NS | 1.01 [1.00-1.01]  *P<*0.0001 | 0.92 [0.88-0.97]  *P =* 0.001447 | 0.96 [0.91-1.02]  NS | 1.13 [1.06-1.21]  *P =* 0.000357 | 0.90  [0.82-0.98]  *P =* 0.015024 | 0.1160703 |
|  | AA vs. GG | 0.98 [0.88-1.08]  NS |  |  |  |  |  |  |
| Hypertension (SBP ≥ 140 mmHg and/or DBP ≥ 90 mmHg) | AA vs. AG | 1.06 [0.999-1.13]  NS | 1.01 [1.01-1.01]  *P<*0.0001 | 0.96 [0.90-1.02]  NS | 1.13 [1.05-1.21]  *P<*0.0001 | 1.08 [0.99-1.17]  NS | 0.82  [0.74-0.91]  *P =* 0.000161 | 0.1411894 |
|  | AA vs. GG | 0.99 [0.87-1.12]  NS |  |  |  |  |  |  |
| High Cholesterol (≥ 240 mg/dL) | AA vs. AG | 0.99 [0.95-1.04]  NS | 1.00 [1.00-1.00]  *P =* 0.000409 | 0.95 [0.92-0.99]  *P =* 0.014225 | 1.01 [0.96-1.06]  NS | 0.97 [0.92-1.02]  NS | 0.98  [0.92-1.05]  NS | 0.04622463 |
|  | AA vs. GG | 1.00 [0.92-1.09]  NS |  |  |  |  |  |  |
| High LDL (serum LDL ≥140 mg/dL) | AA vs. AG | 0.99 [0.92-1.06]  NS | 1.01 [1.01-1.01]  *P<*0.0001 | 0.89 [0.83-0.95]  *P<*0.0001 | 1.03 [0.95-1.11]  NS | 1.06 [0.97-1.16]  NS | 0.90  [0.80-1.01]  NS | 0.1343167 |
|  | AA vs. GG | 1.00 [0.86-1.16]  NS |  |  |  |  |  |  |

BMI, body mass index; DBP, diastolic blood pressure; LDL, low-density lipoprotein; SBP, systolic blood pressure
