# Supplementary material for: Polymorphisms associated with a tropical climate and root crop diet induce susceptibility to metabolic and cardiovascular diseases in Solomon Islands
Source: PLoS One. 2017 Mar 2;12(3):e0172676. doi: 10.1371/journal.pone.0172676 (PMC5333831; doi:10.1371/journal.pone.0172676)
Supplement: S10 Table — (DOCX) [file pone.0172676.s010.docx]

S10 Table. The effects of the variant allele of rs174570 on the occurrence of diseases

|  | Polymorphism | | Age | Sex  (Female = 0;  Male = 1) | Population difference | | Intercept | Nagelkerke *R^2^* |
| --- | --- | --- | --- | --- | --- | --- | --- | --- |
|  |  |  |  |  | Munda = 1 | Ravaki = 1 |  |  |
| Overweight (BMI ≥ 25 kg/m^2^) | CC vs. CT | 0.74 [0.38-1.41]  NS | 1.01 [1.00-1.03]  *P =* 0.049878 | 0.32 [0.22-0.47]  *P<*0.0001 | 2.19 [1.41-3.42]  *P =* 0.000498 | 7.99 [4.62-14.18]  *P<*0.0001 | 0.56  [0.23-1.31]  NS | 0.2351135 |
|  | CC vs. TT | 0.78 [0.40-1.51]  NS |  |  |  |  |  |  |
| Diabetes (serum glucose ≥110 mg/dL) | CC vs. CT | 1.01 [0.93-1.10]  NS | 1.01 [1.00-1.01]  *P<*0.0001 | 0.92 [0.88-0.97]  *P =* 0.00137 | 0.96 [0.90-1.02]  NS | 1.12 [1.04-1.19]  *P =* 0.00143 | 0.91  [0.81-1.01]  NS | 0.1206967 |
|  | CC vs. TT | 0.97 [0.89-1.05]  NS |  |  |  |  |  |  |
| Hypertension (SBP ≥ 140 mmHg and/or DBP ≥ 90 mmHg) | CC vs. CT | 0.94 [0.85-1.04]  NS | 1.01 [1.01-1.01]  *P<*0.0001 | 0.95 [0.90-1.01]  NS | 1.11 [1.04-1.20]  *P =* 0.00329 | 1.03 [0.95-1.12]  NS | 0.92  [0.81-1.05]  NS | 0.1417076 |
|  | CC vs. TT | 0.90 [0.81-0.997]  *P =* 0.04512 |  |  |  |  |  |  |
| High Cholesterol (≥ 240 mg/dL) | CC vs. CT | 1.00 [0.94-1.07]  NS | 1.00 [1.00-1.00]  *P =* 0.000363 | 0.95 [0.92-0.99]  *P =* 0.015581 | 1.01 [0.97-1.06]  NS | 0.98 [0.93-1.03]  NS | 0.96  [0.88-1.05]  NS | 0.04900734 |
|  | CC vs. TT | 1.03 [0.96-1.10]  NS |  |  |  |  |  |  |
| High LDL (serum LDL ≥140 mg/dL) | CC vs. CT | 1.04 [0.93-1.17]  NS | 1.01 [1.01-1.01]  *P<*0.0001 | 0.89 [0.83-0.95]  *P =* 0.000892 | 1.03 [0.95- 1.17]  NS | 1.07 [0.97-1.18]  NS | 0.86  [0.73-0.999]  *P =* 0.049123 | 0.135274 |
|  | CC vs. TT | 1.05 [0.93-1.18]  NS |  |  |  |  |  |  |

BMI, body mass index; DBP, diastolic blood pressure; LDL, low-density lipoprotein; SBP, systolic blood pressure
