# Supplementary material for: Polymorphisms associated with a tropical climate and root crop diet induce susceptibility to metabolic and cardiovascular diseases in Solomon Islands
Source: PLoS One. 2017 Mar 2;12(3):e0172676. doi: 10.1371/journal.pone.0172676 (PMC5333831; doi:10.1371/journal.pone.0172676)
Supplement: S11 Table — (DOCX) [file pone.0172676.s011.docx]

S11 Table. The effects of the variant allele of rs185819 on the occurrence of diseases

|  | Polymorphism | | Age | Sex  (Female = 0;  Male = 1) | Population difference | | Intercept | Nagelkerke *R^2^* |
| --- | --- | --- | --- | --- | --- | --- | --- | --- |
|  |  |  |  |  | Munda = 1 | Ravaki = 1 |  |  |
| Overweight (BMI ≥ 25 kg/m^2^) | CC vs. CT | 1.08 [0.66-1.77]  NS | 1.01 [0.99-1.03]  NS | 0.33 [0.22-0.49]  *P<*0.0001 | 2.26 [1.46-3.53]  *P =* 0.000301 | 6.23 [3.47-11.45]  *P<*0.0001 | 0.37 [0.18-0.75]  *P =* 0.00637 | 0.2422533 |
|  | CC vs. TT | 1.73 [0.96-3.13] NS |  |  |  |  |  |  |
| Diabetes (serum glucose ≥110 mg/dL) | CC vs. CT | 0.98 [0.92-1.05]  NS | 1.01 [1.00-1.01]  *P<*0.0001 | 0.92 [0.88-0.97]  *P =* 0.00114 | 0.96 [0.91-1.02]  NS | 1.15 [1.07-1.24] *P =* 0.00017 | 0.91 [0.83-1.00]  *P =* 0.04774 | 0.1176077 |
|  | CC vs. TT | 0.96 [0.89-1.04]  NS |  |  |  |  |  |  |
| Hypertension (SBP ≥ 140 mmHg and/or DBP ≥ 90 mmHg) | CC vs. CT | 0.95 [0.88-1.03]  NS | 1.01 [1.01-1.01]  *P<*0.0001 | 0.95 [0.90-1.01]  NS | 1.12 [1.05-1.20]  *P =* 0.0014 | 1.04 [0.95-1.14]  NS | 0.87 [0.78-0.98]  *P =* 0.0172 | 0.1394266 |
|  | CC vs. TT | 1.01 [0.92-1.11]  NS |  |  |  |  |  |  |
| High Cholesterol (≥ 240 mg/dL) | CC vs. CT | 1.04 [0.99-1.10]  NS | 1.00 [1.00-1.00] *P =* 0.000213 | 0.95 [0.92-0.99]  *P =* 0.013867 | 1.01 [0.96-1.05]  NS | 0.99 [0.93-1.05] | 0.96 [0.89-1.03]  NS | 0.05579026 |
|  | CC vs. TT | 0.99 [0.94-1.06]  NS |  |  |  |  |  |  |
| High LDL (serum LDL ≥140 mg/dL) | CC vs. CT | 0.97 [0.88-1.06]  NS | 1.01 [1.01-1.01] *P<*0.0001 | 0.88 [0.82-0.95]  *P =* 0.000514 | 1.02 [0.95-1.11]  NS | 1.10 [0.99-1.22]  NS | 0.93 [0.81-1.05]  NS | 0.1377181 |
|  | CC vs. TT | 0.93 [0.83-1.03]  NS |  |  |  |  |  |  |

BMI, body mass index; DBP, diastolic blood pressure; LDL, low-density lipoprotein; SBP, systolic blood pressure
