# Supplementary material for: Polymorphisms associated with a tropical climate and root crop diet induce susceptibility to metabolic and cardiovascular diseases in Solomon Islands
Source: PLoS One. 2017 Mar 2;12(3):e0172676. doi: 10.1371/journal.pone.0172676 (PMC5333831; doi:10.1371/journal.pone.0172676)
Supplement: S12 Table — (DOCX) [file pone.0172676.s012.docx]

S12 Table. The effects of the variant allele of rs2237892 on the occurrence of diseases

|  | Polymorphism | | Age | Sex  (Female = 0;  Male = 1) | Population difference | | Intercept | Nagelkerke *R^2^* |
| --- | --- | --- | --- | --- | --- | --- | --- | --- |
|  |  |  |  |  | Munda = 1 | Ravaki = 1 |  |  |
| Overweight (BMI ≥ 25 kg/m^2^) | CC vs. CT | 0.94 [0.62-1.43]  NS | 1.01 [1.00-1.03]  *P =* 0.037456 | 0.31 [0.21-0.46]  *P<*0.0001 | 2.14 [1.37-3.35]  *P<*0.0001 | 8.32 [4.91-14.50]  *P<*0.0001 | 0.42  [0.22-0.79]  *P =* 0.007856 | 0.2422533 |
|  | CC vs. TT | 1.40 [0.77-2.57]  NS |  |  |  |  |  |  |
| Diabetes (serum glucose ≥110 mg/dL) | CC vs. CT | 0.97 [0.92-1.02]  NS | 1.01 [1.00-1.01]  *P<*0.0001 | 0.92 [0.88-0.97]  *P =* 0.00188 | 0.97 [0.92-1.03]  NS | 1.13 [1.06-1.21]  *P =* 0.00014 | 0.91  [0.84-0.99]  *P =* 0.02784 | 0.119335 |
|  | CC vs. TT | 0.95 [0.88-1.03]  NS |  |  |  |  |  |  |
| Hypertension (SBP ≥ 140 mmHg and/or DBP ≥ 90 mmHg) | CC vs. CT | 1.05 [0.98-1.12]  NS | 1.01 [1.01-1.01]  *P<*0.0001 | 0.95 [0.90-1.01]  NS | 1.11 [1.03-1.19]  *P =* 0.004498 | 1.06 [0.98-1.14]  NS | 0.83  [0.75-0.91]  *P<*0.0001 | 0.1380385 |
|  | CC vs. TT | 1.05 [0.96-1.15]  NS |  |  |  |  |  |  |
| High Cholesterol (≥ 240 mg/dL) | CC vs. CT | 0.99 [0.95-1.04]  NS | 1.00 [1.00-1.00]  NS | 0.95 [0.91-0.99]  NS | 1.01 [0.96-1.05]  NS | 0.97 [0.92-1.02]  NS | 0.97  [0.91-1.04]  NS | 0.05355711 |
|  | CC vs. TT | 1.05 [0.99-1.12]  NS |  |  |  |  |  |  |
| High LDL (serum LDL ≥140 mg/dL) | CC vs. CT | 0.99 [0.92-1.07]  NS | 1.01 [1.01-1.01]  *P<*0.0001 | 0.88 [0.82-0.95]  *P =*0.000494 | 1.02 [0.94-1.11]  NS | 1.07 [0.98-1.17]  NS | 0.89  [0.79-1.00]  *P =* 0.054976 | 0.1353815 |
|  | CC vs. TT | 1.05 [0.94-1.17]  NS |  |  |  |  |  |  |

BMI, body mass index; DBP, diastolic blood pressure; LDL, low-density lipoprotein; SBP, systolic blood pressure
