# Supplementary material for: Polymorphisms associated with a tropical climate and root crop diet induce susceptibility to metabolic and cardiovascular diseases in Solomon Islands
Source: PLoS One. 2017 Mar 2;12(3):e0172676. doi: 10.1371/journal.pone.0172676 (PMC5333831; doi:10.1371/journal.pone.0172676)
Supplement: S13 Table — (DOCX) [file pone.0172676.s013.docx]

S13 Table. The effects of the variant allele of rs2722425 on the occurrence of diseases

|  | Polymorphism | | Age | Sex  (Female = 0;  Male = 1) | Population difference | | Intercept | Nagelkerke *R^2^* |
| --- | --- | --- | --- | --- | --- | --- | --- | --- |
|  |  |  |  |  | Munda = 1 | Ravaki = 1 |  |  |
| Overweight (BMI ≥ 25 kg/m^2^) | GG vs. GA | 0.79 [0.52-1.19]  NS | 1.02 [1.00-1.03]  *P =* 0.03327 | 0.33 [0.22-0.48]  *P<*0.0001 | 2.18 [1.41-3.39]  *P =* 0.00051 | 9.58 [5.51-17.21]  *P<*0.0001 | 0.45 [0.24-0.84]  *P =* 0.01222 | 0.2436032 |
|  | GG vs. AA | 0.41 [0.15-1.09]  NS |  |  |  |  |  |  |
| Diabetes (serum glucose ≥110 mg/dL) | GG vs. GA | 0.99 [0.94-1.05]  NS | 1.01 [1.00-1.01]  *P<*0.0001 | 0.92 [0.88-1.12]  *P =* 0.001888 | 0.96 [0.91-1.02]  NS | 1.13 [1.06-1.21]  *P =* 0.000205 | 0.89 [0.82-0.97]  *P =* 0.006360 | 0.1152555 |
|  | GG vs. AA | 0.99 [0.87-1.12]  NS |  |  |  |  |  |  |
| Hypertension (SBP ≥ 140 mmHg and/or DBP ≥ 90 mmHg) | GG vs. GA | 1.02 [0.96-1.09]  NS | 1.01 [1.01-1.01]  *P<*0.0001 | 0.95 [0.90-1.01]  NS | 1.12 [1.04-1.20]  *P =* 0.00177 | 1.05 [0.97-1.13]  NS | 0.84 [0.77-0.93]  *P<*0.0001 | 0.1379415 |
|  | GG vs. AA | 1.12 [0.96-1.29]  NS |  |  |  |  |  |  |
| High Cholesterol (≥ 240 mg/dL) | GG vs. GA | 1.05 [1.01-1.10]  *P =* 0.014309 | 1.00 [1.00-1.00]  *P =* 0.000621 | 0.95 [0.91-0.99]  *P =* 0.009948 | 1.01 [0.97-1.06]  NS | 0.96 [0.91-1.01]  NS | 0.97 [0.91-1.03]  NS | 0.05980959 |
|  | GG vs. AA | 1.08 [0.98-1.19]  NS |  |  |  |  |  |  |
| High LDL (serum LDL ≥140 mg/dL) | GG vs. GA | 1.12 [1.04-1.20]  *P =* 0.002849 | 1.01 [1.01-1.01]  *P<*0.0001 | 0.88 [0.83-0.95]  *P =*0.000497 | 1.03 [0.95-1.12]  NS | 1.05 [0.95-1.15]  NS | 0.87 [0.77-0.97]  *P =* 0.012277 | 0.1496188 |
|  | GG vs. AA | 1.07 [0.90-1.27]  NS |  |  |  |  |  |  |

BMI, body mass index; DBP, diastolic blood pressure; LDL, low-density lipoprotein; SBP, systolic blood pressure
